# The Relationship between Stroop and Stop-Signal Measures of Inhibition in Adolescents: Influences from Variations in Context and Measure Estimation

**DOI:** 10.1371/journal.pone.0101356

**Published:** 2014-07-03

**Authors:** Kiat Hui Khng, Kerry Lee

**Affiliations:** 1 Centre for Research in Pedagogy and Practice, National Institute of Education, Nanyang Technological University, Singapore, Singapore; 2 Centre for Research in Pedagogy and Practice/Office of Educational Research/Psychological Studies Academic Group, National Institute of Education, Nanyang Technological University, Singapore, Singapore; University of Verona, Italy

## Abstract

The Stroop and stop-signal tasks are commonly used to index prepotent response inhibition in studies of cognitive development and individual differences. Inhibitory measures from the two tasks have been derived using a variety of methods. Findings of low inter-correlations amongst these measures have been interpreted as evidence for different kinds of inhibitory functions. Our previous study found Stroop and stop-signal accuracy measures to be uncorrelated and they loaded on different inhibitory components in a principal component analysis. The present study examined whether this finding is replicated across different task contexts, derived measures, and methods of derivation. Adolescents (N = 247) were administered a number-quantity Stroop and word and number stop-signal tasks. For each stop-signal task, inhibitory efficiency was estimated using a stop-signal reaction time measure estimated with the central versus the integration methods. For the Stroop interference task, inhibitory efficiency was indexed by reaction time measures (including inverse efficiency scores) generated from difference scores and regression residuals, and delta-plot slopes. The reaction time measures from the two tasks were generally not correlated. The only exception was that Stroop inhibitory *ability*, indexed by Stroop errors, was related to stop-signal inhibitory *efficiency*, indexed by stop-signal reaction time. These findings are consistent with previous findings suggesting that measures from the Stroop and stop-signal tasks are influenced by different underlying processes. The impact of variations in dependent measure derivation on the resulting reliabilities of Stroop and stop-signal measures and on observed correlations between them were examined. Variables that may have contributed to the null findings are discussed.

## Introduction

Inhibition, the ability to suppress or resist irrelevant information, processes or responses, is a core function required for the control of thought and action. Changes in the efficiency of various cognitive abilities–from working memory to intelligence–have been attributed to the development or integrity of inhibitory control [1].

Inhibition is often studied as resistance to interference [1] and is commonly viewed as a family of related functions [2]. However, there are differences in opinions on how the various functions are related or constituted. Nigg [3], for example, outlined eight kinds of inhibition including behavioral and cognitive inhibition and interference control. Lustig, Hasher and Zacks [4] differentiated among access, deletion and restraint inhibitory functions. In addition to definitional problems, advancements in the field are hampered by measurement problems, such as the use of complex tasks that require multiple processes in addition to inhibition (e.g., Wisconsin Card Sorting Test or the Tower of Hanoi). Also problematic is the widespread use of subtraction or difference scores for estimating inhibitory efficiency, which tend to show much poorer reliability than their constituent scores [5].

Such measurement and analytical problems make it difficult to interpret findings from different inhibitory tasks. Here, we describe a preliminary study on whether two widely-used tests of inhibition–the Stroop [6] and stop-signal [7] tasks–measure the same type of inhibitory ability. Both tasks are often used to index prepotent response inhibition. However, the extent to which they measure the same construct is unclear.

### The Stroop and Stop-Signal Paradigms

#### Stroop

A typical Stroop task contains two overlapping stimulus-response dimensions [8]. In the classic color-word Stroop task (see [9] for a review), participants are asked to name the ink-color in which a color-word is printed (e.g., RED printed in blue ink). Interference, also known as the Stroop effect, occurs when the relevant (i.e., color-naming) and irrelevant dimensions (i.e., word-reading) lead to overlapping but incongruent responses. Compared to a neutral (e.g., a color patch) or congruent (e.g., RED printed in red) stimulus, naming of the ink-color takes longer and often results in intrusion errors (e.g., answering “red” to RED printed in blue). Facilitation occurs in the congruent condition where the two dimensions lead to compatible responses, resulting in faster and more accurate responses.

Stroop facilitation and interference effects are usually attributed to word-reading being the more practiced and hence more prepotent stimulus-response dimension than color-naming. Accurate performance on incongruent trials is commonly thought to be achieved by selective inhibition dampening the fast automatic activation associated with word-reading, so the slower deliberate route associated with color-naming may be completed [10], [11]. Stroop interference, measured by the difference in latency or accuracy between (a) the incongruent and neutral or (b) incongruent and congruent conditions, is typically taken to reflect inhibitory ability or efficiency.

#### Stop-signal

The stop-signal paradigm requires a rapid and practiced response to a visual stimulus on “go” trials, and the withholding of that response upon the presentation of a stop-signal on a minority of “stop” trials. Success or failure of inhibition is believed to be dependent on the relative finishing times of the stochastically independent “go” and “stop” processes [7]. Measures reflecting rates of successful or failed inhibition on “stop” trials (probability of stopping & commission errors) and covert stopping speed (stop-signal reaction time; SSRT) are commonly used to index inhibitory ability or efficiency.

It should be noted that we limit our scope here to stop-signal tasks based on Logan and Cowan’s paradigm [7]. Such choice-reaction-time tasks typically involve centrally presented stimuli and manual key-press responses, and are commonly used in cognitive psychology to study individual, clinical and developmental differences in the inhibition of responses. Other countermanding paradigms have been used to study the inhibition of saccadic eye or arm reaching movements to peripheral stimuli in both monkeys [12], [13] and humans [14].

### Relationship between the Stroop and Stop-Signal Tasks

Both the Stroop and stop-signal tasks can be seen as requiring the inhibition of a prepotent or well-practiced response. Lustig et al. [4] conceptualized Stroop and stop-signal inhibition as serving a similar restraint function of suppressing strong but inappropriate responses. Findings that performances on the two tasks are correlated with each other or load on a common factor support a common underlying construct [2], [15], [16], [17].

In contrast, there is also evidence suggesting that the two tasks may index different constructs. Stroop and stop-signal measures of inhibition have been found to be uncorrelated or loaded onto different factors [18], [19]; exhibit different developmental trajectories [19], [20]; show different patterns of performance impairment in clinical subgroups (e.g., borderline personality disorder & attention deficit and hyperactivity disorder [21]; dementia of the Alzheimer type [22]), show different outcomes following drug administration [23], [24], and activate diverse, but overlapping brain regions [25], [26], [27]. It has been argued that the two tasks may index different aspects of inhibition–e.g., inhibition of reified/well-entrenched processes in the Stroop versus inhibition of recently learned associations in the stop-signal [19]. Some have even argued that, other than the cancellation of motor responses (e.g., in the stop-signal task), most ‘inhibitory’ phenomena (including the Stroop effect) may be explained by “inhibition-free model(s)” (p.183) [28]. For example, it is possible that Stroop interference may be accounted for by proactive mechanisms such as sustained activation of goal or task representations [29], [30]. More recent models of inhibitory control tend to conceptualize performance on tasks such as the Stroop and stop-signal as a result of both proactive/early-selection mechanisms (e.g., goal maintenance) and reactive/late-correction mechanisms (e.g., interference/conflict resolution), with the emphasis on proactive/reactive control amenable to variations in both person and situational factors (e.g., working memory capacity, age & motivational context) [30], [31], [32], [33], [34].

Empirical findings of a weak (or null) relationship between the two tasks’ measures may thus reflect the engagement of different constructs or different combinations of similar processes. On the other hand, low correlations may also arise from measurement issues. The literature sees a mix of accuracy and reaction time (RT) scores from different task variants, often computed in different ways across studies.

#### Variation in task contexts

Stroop effects can be elicited using a variety of stimuli, including the classic color-word version, but also with stimuli such as numerals and picture-word [9], [35]. Whether Stroop interference is correlated across task variants can depend on the degree of similarity between tasks (e.g., type of stimuli or stimulus-response dimension [27], [35]). Stop-signal tasks vary widely in the primary or “go” task. There are also visual versus auditory versions of the “stop” signal [2], [15]. SSRT has been found to be shorter for auditory compared to visual “stop” signals [36], but is considerably robust across “go” task variations [36], [37].

#### Variation in measures

Inhibitory control in the Stroop task is commonly indexed by Stroop interference reaction time, amongst other measures such as accuracy rates and commission/intrusion errors. Stroop interference RT is calculated equally often as the mean latency difference between incongruent and neutral conditions, and between incongruent and congruent conditions. The neutral control is sometimes preferred over the congruent condition which can be confounded by individual differences in facilitation effects. On the other hand, the congruent condition serves as a closer control for the incongruent condition in terms of stimulus-response dimensional overlap. Some studies have examined interference RT in terms of delta-plots [38] derived by rank-ordering each participant’s RTs for each condition and then plotting the mean interference RT by quantiles. A weak or slow inhibitory process is hypothesized to benefit Stroop performance most at slower RTs, giving an accurate-trials RT distribution a negative skew, and a steeper interference delta slope [11].

Though not conventionally used, the recent years have also seen Stroop interference begin to be examined in terms of inverse efficiency (IE)–an adjusted RT measure derived by dividing RT (e.g., mean RT) by its corresponding percentage accuracy–in a small handful of studies [39], [40], [41]. Conventional RT measures are typically based on accurate trials only. The IE score adjusts RT performance for sacrifices in accuracy that might have been made in favor of speed. A mean RT achieved with high accuracy will have a smaller IE (i.e., is more efficient) than the same RT achieved at the cost of more errors. The hybrid IE score may be especially useful when there are individual or developmental differences in speed-accuracy trade-offs, in which case accuracy and RT data can show different patterns of results [42].

One of the most prevalent measures of inhibitory control in the stop-signal task is the SSRT [43]. The SSRT is an estimate of stopping or inhibition speed and is derived by subtracting from a measure of “go” RT, a measure of the stop-signal delay (SSD)–the stimulus-onset asynchrony between “go” and “stop” stimuli. However, SSD is determined differently across studies and can take the form of a single fixed SSD, average of multiple fixed SSDs, or SSD tracking [44]. Even when the same SSD is used, there are differences in the way in which the SSRT is computed. An estimate commonly used is the SSRT central (SSRT_central_), computed at the central SSD where the race between “go” and “stop” processes ends in a tie and the success/failure rate of inhibition is 50%. The central SSD is often estimated with a tracking algorithm [45] that dynamically adjusts the SSD according to performance on the previous “stop” trial. That is, following each successful “stop”, the likelihood of successful inhibition at the next “stop” trial would be decreased by delaying the onset of the stop-signal.

SSRT_central_ is reportedly the most accurate and reliable estimate of stop-signal inhibitory efficiency when achieved response rates are around 50% [44]. However, it can over-estimate SSRT when response rates deviate from 50%, for example, when participants engage in strategic response slowing in anticipation of the “stop” stimuli, or when the RT distribution is positively skewed [43]. In this case, computing SSRT using the integration method (SSRT_integration_) has been argued to be more robust as it takes into account the actual response rate achieved [46]. This method involves rank-ordering “go” RTs and subtracting the SSD at the actual achieved response rate from the “go” RT value at the percentile corresponding to the achieved response rate [7]. However, SSRT_integration_ tends to be underestimated when there is gradual response slowing over trials. In the case that subjects exhibiting slowing cannot be removed from analysis, SSRT_integration_ can be calculated as an average over smaller blocks of trials to yield a more accurate estimation of SSRT [43].

Less widely used measures of stop-signal inhibition include commission errors, probability of inhibition, and the inhibition function curve [47]. Such measures are however, limited to paradigms that employ fixed stop-signal delays (SSD) as they will be artificially influenced by tracking algorithms.

Because different measures may emphasize the influence of different processes underlying the Stroop or stop-signal task, different findings can be expected across studies that used different measures. To our knowledge, only one study has specifically examined how variations in calculating SSRT can affect its relationship with other measures [46]. None has compared variations in calculating Stroop interference measures. It is an aim of the present study to examine if inconsistent findings on the relationship between Stroop and stop-signal measures may be due in part to variations in how dependent measures were derived.

### The Present Study

The present study explored the relationship between Stroop and stop-signal inhibition using a variety of derived measures. In a previous study, we examined the relationship amongst six inhibitory tasks and how they predicted algebra word problem solving performance in young adolescents. Commission errors from a numerical Stroop task and a word-categorization stop-signal task were not significantly correlated and loaded on different inhibitory constructs [19]. The present study examined whether significant correlations would be found if RT based measures were used.

To maintain comparability with our earlier study, the numerical Stroop and word-categorization stop-signal tasks from the previous study [19] were administered to a similar sample of adolescents. Although previous studies suggest that differences in the “go” task has little effects on the estimation of the SSRT, to attenuate differences that may result from the use of different stimuli, we administered an additional number-categorization stop-signal task. To examine the impact of choice of measures, we examined SSRT and Stroop interference difference scores, delta-plot slopes, and commission errors. Given the low reliability associated with subtraction scores, we also computed inhibitory measures using a residualised method. To examine the impact of variations in computation of measures, we examined SSRTs calculated using two different methods–SSRT_central_ and SSRT_integration_, and Stroop interference RT and IE scores calculated with both neutral and congruent baselines.

## Methods

### Ethics Statement

At the time this study was conducted, there was not an ethics review board at our university. For this reason, we followed convention and sought approval from the school authorities. A letter inviting schools’ participation was first sent to the schools via email. The invitation letter described the aims and target sample of the study, the tasks that the participants would be administered, and the time commitment required from participating schools and students. It also stated that the privacy of the children’s data will be protected; only summary data will be reported at the end of the study. An information sheet and consent form was sent out to parents of potential participants through the participating schools. The information sheet contained similar information as the letter of invitation to schools. Additionally, the voluntary nature of participation was emphasized. Parents who consented to their child’s participation signed and returned the consent form through the child’s school. Verbal assent was obtained from the participants on the day of data collection. Participants were free to withdraw from the study at any point in time. All research was conducted in compliance with the “Helsinki Declaration” [48], and the Singapore Psychological Society’s “Code of Professional Ethics” [49].

### Participants and Procedure

A total of 247 Secondary 2 students (Grade 8) from 6 Singapore schools participated in the study. Students in secondary schools are streamed into different academic streams based on their performances in a national examination conducted at the end of primary school. Students from each of the four academic streams were included in proportions corresponding to the national distribution.

Participants were tested on computerized versions of a Stroop and two stop-signal tasks in a single one-hour session in small groups in their school computer laboratory. Order of task administration was counterbalanced across participants with rest breaks between blocks and tasks.

Seven students were excluded from the final dataset because of missing or corrupted data. Data from 62 students were further excluded for failing to demonstrate above-chance performance (at least 66% accuracy) on non-inhibitory/baseline trials (Stroop neutral and stop-signal “go” trials). The tasks used in this study were very simple choice reaction time tasks that have been successfully used with even much younger children [50]. Poor performance–despite demonstrating adequate ability on practice trials–is likely to indicate a lack of engagement with the task rather than a lack of ability. Disengaged participants might have become bored of the simple, monotonous tasks, repeated over a large number of trials. The integrity and reliability of data confounded by possible random key-presses would have been compromised and non-reflective of participants’ true ability. The performance criterion for data screening was thus applied. Although the possibility of genuine inability or difficulty performing the task cannot be ruled out, competent performances on practice trials suggest this to be highly unlikely. An examination of the distribution of students in this category revealed a relationship with academic stream (*χ^2^* = 41.10, *df* = 3, *p*<0.001). Most of the excluded students were from the lower academic streams who exhibited less cooperative behavior. The proportion of participants excluded for failing the performance criterion were, from the top to bottom streams, 0%, 20%, 35% and 69%. That is, none of the students from the top academic stream had to be excluded; 69% of the students from the lowest academic stream had to be excluded. The final dataset comprised 178 students (82 boys, mean age = 14.00, range = 13.15–15.89, *SD = *0.47).

As our previous findings suggest that the Stroop and stop-signal measures may be uncorrelated, a power analysis was conducted to estimate the sample size needed to minimize Type II error [51]. The present sample had approximately 80% power in detecting a correlation as small as *r = *.18 (the correlation found in [16]), at α = 0.05 (one-tailed), and 99% power in detecting a moderate-sized correlation (*r = *.30; [52]).

### Materials

**Figure 1:**
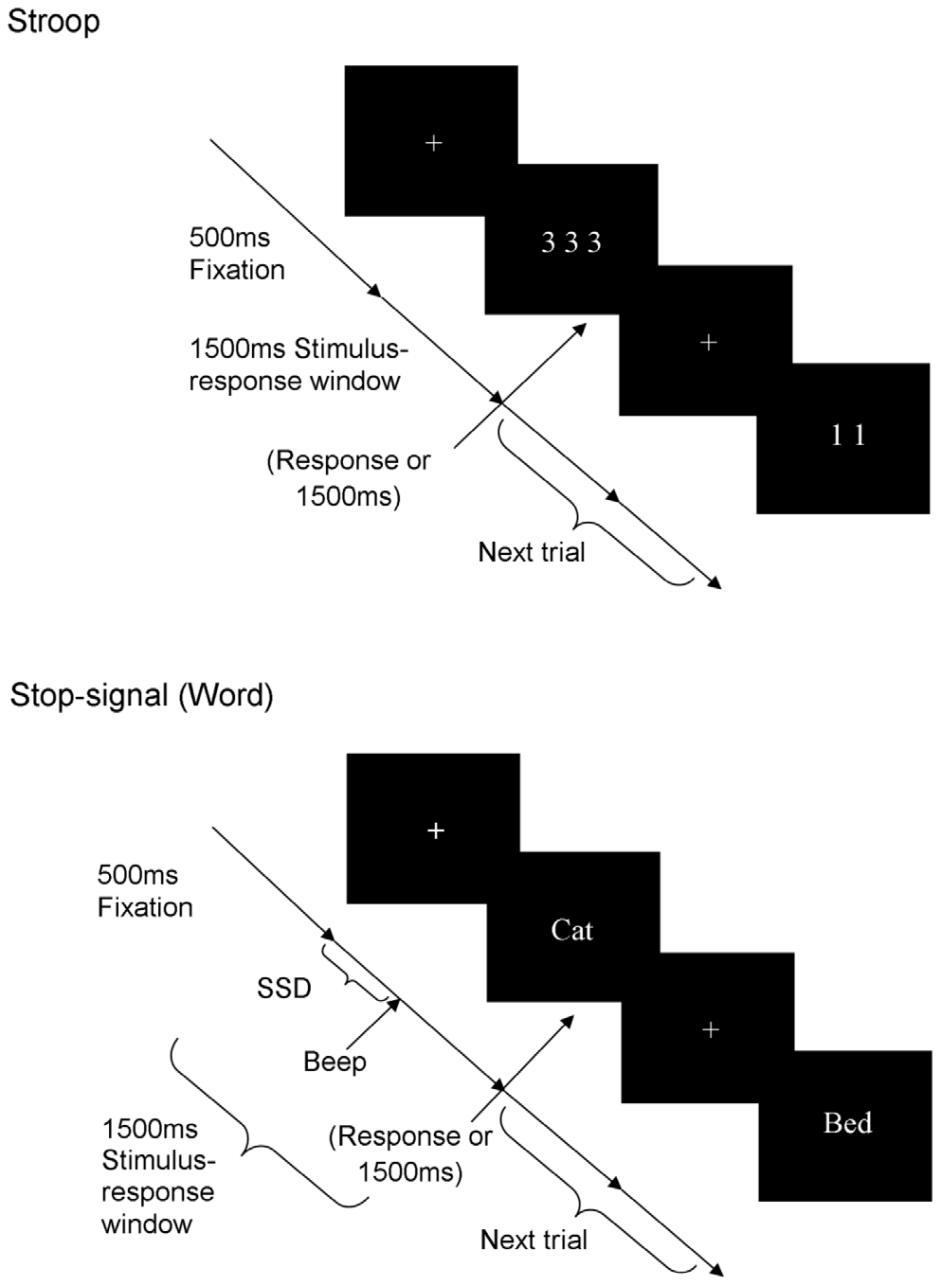
The time-course of events presented in the Stroop and stop-signal tasks. Sample stimuli shown. SSD, stop-signal delay.

The time-course of events presented in the Stroop and stop-signal tasks are presented in Figure 1.

#### Number-quantity Stroop

On each trial, one to four of the same stimuli were presented. Participants indicated *how many* stimuli were presented by pressing the 1, 2, 3, or 4 keys. Participants were instructed to respond as quickly as they can without making errors. Stimuli were asterisks in the neutral condition and the Arabic numeral 1 to 4 in the congruent and incongruent conditions. In the congruent condition, the numeral corresponded to the number of times it is displayed (e.g., “4444”). There was a mismatch in the incongruent condition (e.g., “222”). Participants completed a practice block of 12 trials before 4 blocks of 24 trials. Each block contained equal proportions of each condition in a randomized order.

Dependent measures were: number of intrusion errors on incongruent trials and Stroop interference RT difference scores, residual scores and delta-plot slope coefficients calculated using congruent and neutral baselines. Difference scores were derived by subtracting the mean reaction time of the respective baseline condition from the mean reaction time of the incongruent condition. Residual scores were derived from regressing mean incongruent on baseline mean reaction times. To derive the delta-plot slope coefficient, each participant’s RTs were rank-ordered (from fastest to slowest) by condition and split into 5 quantile bins. Interference RT difference score was calculated for each bin and the slope coefficient is based on the slope for the line of best fit. A parallel set of Stroop interference IE scores was also calculated where applicable (difference and residual scores). These scores were derived the same way as their RT counterparts, using each condition’s IE instead of mean RT. The IE score was calculated for the congruent, neutral and incongruent conditions by dividing each condition’s mean RT with its respective percentage accuracy.

#### Word and number stop-signal

In the word version, participants were instructed to categorize each word displayed as animal or non-animal by key-press, except on trials where they heard a beep (“stop” trials). In the number version, they categorized numerical stimuli as odd or even. Participants were instructed to respond as quickly as they can without making errors. SSDs were adjusted by a staircase tracking algorithm [45] with a start value of 250 ms and a step-size of 50 ms. For practice, participants completed a block of 24 non-signal trials followed by a block of 24 signal trials. Non-signal blocks contained only “go” trials. A beep sounded on 25% of trials in signal blocks. Actual trials comprised a non-signal block of 48 trials and four signal blocks of 100 trials. SSRT_central_ was derived by subtracting from the mean “go” RT, the SSD at which the rate of responding was.5. SSRT_integration_ was derived by first rank-ordering each participant’s “go” RTs. The SSD at the actual achieved rate of responding was then subtracted from the RT value at the percentile corresponding to the achieved response rate [7]. The alternative, block-wise SSRT_integration_ measure suggested by [43]–derived by averaging SSRT_integration_ estimates derived from smaller blocks–was considered in the event that substantial response slowing was observed.

## Results

RT measures were computed from accurate trials only. Data from trials with RT less than 200 ms (anticipatory responses) were excluded. Individual RTs below or exceeding individual mean +/−3 *SD*s for each condition were then replaced with the 3 *SD* values to minimize disproportionate influences from outliers. Outliers on the group level were removed from further analyses. Less than 3% of the data were affected by these procedures.

Descriptives for each task and condition can be found in Table 1.

**Table 1:** Means and Standard Deviations of Component Measures.

	Stroop			Stop-signal	
Variable	Congruent	Neutral	Incongruent	Word-Go	Number-Go
Reaction time	598 (84)	632 (93)	665 (95)	686 (111)	624 (102)
Inverse efficiency	632 (103)	678 (105)	825 (163)	–	–
Accuracy	95 (7)	93 (6)	82 (11)	92 (5)	93 (5)
Response rate	–	–	–	52 (7)	53 (7)

*Note.* Reaction time and Inverse efficiency in ms; Accuracy and Response rate in %.

Figures rounded to nearest whole number.

### Stop-Signal Tracking

The stop-signal tracking worked well generally, with mean rates of responding at.53 and.52 for the number and word stop-signal tasks, respectively. The response rate (RR) of a few participants did deviate substantially from 50%, with a range of .43 to .82 (number stop-signal) and .38 to .82 (word stop-signal). However, most of the deviations from the central range (.40 to .60; [43]) were in the upper end which suggested increased inhibition failure due to fast responding (high RR) rather than decreased inhibition failure due to strategic response slowing (low RR). The alternative block-wise integration method was thus not adopted as its advantage in the case of fast, rather than slow responding is unknown. Instead, a parallel set of correlations was conducted with the data from these participants (RR <.40 or RR >.60; *N* = 12) removed. Individual correlation coefficients differed from.00 to.06 between the two datasets. As the resulting pattern of correlations was similar to that obtained with the full dataset, analyses based on the full dataset are reported below.

### Correlations

Corrected for multiple comparisons, none of the SSRTs were significantly correlated with any of the Stroop interference measures (at *α* = .05, one-tailed). Number of Stroop errors was the only Stroop measure that correlated with SSRTs (significantly with the number and marginally with the word version; see Table 2 for correlations and descriptives). Marginally significant correlations (i.e., significant before correction for multiple comparisons) were observed between SSRT and several Stroop interference IE measures. Overall, the highest correlation (*r* = .25, marginally significant) between SSRT and Stroop interference was observed when SSRT was estimated by the central method using a number categorization task and Stroop interference was measured in terms of the difference in inverse efficiency between incongruent and congruent conditions. The lowest correlation (*r* = .00, non-significant) was observed when SSRT was estimated by the integration method using a number categorization task and Stroop interference was measured in terms of a residualized RT score with a congruent baseline.

**Table 2:** Means, Standard Deviations and Bivariate Correlations between Measures of Inhibition.

	Variable	*Mean (SD)*	1	2	3	4	5	6	7	8	9	10	11	12	13	14
1	StroopRT_DIFF_N_	36 (38)														
2	StroopRT_DIFF_C_	69 (44)	.60*													
3	StroopRT_RES_N_	0.00 (.08)	1.00^a^ *	.62*												
4	StroopRT_RES_C_	0.00 (.08)	.60*	1.00^a^ *	.62*											
5	StroopRT_SLOPE_N_	.18 (.05)	.33*	.28*	.32*	.29*										
6	StroopRT_SLOPE_C_	.33 (.05)	.09	.38*	.08	.40*	.38*									
7	StroopIE_DIFF_N_	150 (116)	.34*	.26*	.33*	.26*	.17†	.02								
8	StroopIE_DIFF_C_	195 (123)	.23†	.49*	.24†	.49*	.16†	.17†	.84*							
9	StroopIE_RES_N_	0.00 (1.00)	.35*	.23†	.33*	.24†	.18†	.04	1.00^a^ *	.82*						
10	StroopIE_RES_C_	0.00 (1.00)	.23†	.49*	.24†	.49*	.16†	.18†	.83*	1.00^a^ *	.81*					
11	StroopERR	5 (3)	−.09	.01	−.10	0.02	0.02	0.06	.75*	.77*	.75*	.77*				
12	Word-SSRT_central_	292 (89)	−.09	.04	−.08	0.03	0.00 a	0.04	.12	.16†	.10	.15†	.20†			
13	Word-SSRT_integration_	277 (121)	−.13	.05	−.11	0.04	0.06	.08	.14†	.20†	.11	.19†	.22†	.89*		
14	Number-SSRT_central_	280 (90)	−.12	.06	−.10	0.05	−0.05	−0.01	.18†	.25†	.15†	.24†	.29*	.71*	.63*	
15	Number-SSRT_integration_	268 (115)	−.17	.01	−.15	0.00 a	−0.08	0.03	.11	.19†	.08	.18†	.27*	.66*	.62*	.91*

*Note.* Stroop: RT, reaction-time-based scores; IE, inverse-efficiency-based scores; DIFF, difference scores; RES, regression residual scores; SLOPE, delta-plot slope coefficient; subscript N, Neutral baseline; subscript C, Congruent baseline; ERR, number of Stroop intrusion errors. Stop-signal: SSRT_central_, SSRT central; SSRT_integration_, SSRT integration.

† *p*<0.05, one-tailed;

*significant after Bonferroni correction.

a rounding.

To examine the extent to which the variation and low inter-task correlations can be attributed to differences across measures and item reliability, additional analyses were conducted. SSRTs from the same task estimated by different methods were highly correlated (*r = *.89–.91). Variations in method of derivation affected the Stroop measures more. For instance, Stroop delta-plot slope coefficients derived from neutral and congruent baselines were only moderately correlated (*r = *.38). Variations in task context also affected the Stroop measures more than SSRT. Consistent with previous findings, SSRTs were strongly correlated across “go” task variations, for both SSRT_central_ (*r = *.71) and SSRT_integration_ (*r = *.62). On the other hand, different RT measures of the Stroop task were less strongly and consistently correlated with one another (*r* = −.10–.40). The only exceptions were the correlations between Stroop interference difference and residual scores (*r = *.60–.1.00), which were expectedly high since they both reflected incongruent RT controlling for baseline RT. These correlations were even stronger when based on IE measures (*r = *.81–.1.00).

### Reliability

Low observed correlations may result from poor reliabilities instead of lack of a true relationship [2], [53]. Split-half reliabilities adjusted with the Spearman-Brown formula were computed for the mean reaction time of each condition of the Stroop task, using odd and even blocks. Due to the nature of the stop-signal tracking, split-half reliabilities for the SSRTs and “go” mean reaction times were calculated based on the first and second halves of trials. Reliabilities for the Stroop difference (equation 1) and regression residual scores (equation 2) were calculated with the conventional formulae adjusting for the correlation between components [54]. For the IE-based difference and regression residual scores, reliabilities of the component IE scores were first calculated with the formulae for ratio scores (equation 3) [55]:
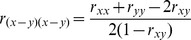
(1)

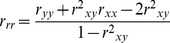
(2)

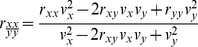
(3)


Due to the high inter-correlations amongst incongruent, neutral and congruent mean reaction times, reliabilities of the Stroop RT-based difference and residual scores were very poor (*r* = .02–.25; Table 3). In comparison, the component RTs that contributed to the computation of these scores (incongruent, neutral and congruent mean reaction time) had high reliabilities (*SB_2_ r = *.91–.93). On the other hand, despite being derived measures, the incongruent, neutral and congruent IEs had comparably high reliability (*r = *.91–.93). This may be attributed to the low correlations between each condition’s RT and accuracy. Similarly, the lower inter-correlations amongst IE scores meant that the reliabilities of their difference and residual scores (*r* = .72–.77) were higher than the RT-based measures, even though they still fell short of conventions for adequacy. Compared to the Stroop measures, the SSRTs showed much higher reliability (*SB_2_ r = *.88–.91). Reliability of the “go” mean reaction times were also high (*SB_2_ r = *.94–.96).

**Table 3:** Reliability Statistics of Stroop and Stop-signal Measures and Correlations between Components.

	Variable	*SB_2_ r*/*r*	1	2	3	4	5	6	7	8
1	StroopMRT_C	.91								
2	StroopMRT_N	.93	.92*							
3	StroopMRT_I	.91	.89*	.92*						
4	StroopIE_C	.91	.82**	.72*	.68*					
5	StroopIE_N	.93	.82*	.90*	.82*	.79*				
6	StroopIE_I	.91	.47*	.51*	.56*	.66*	.71*			
7	StroopAcc_C	.74	.08	.13	.14	−.49*	−.13	−.42*		
8	StroopAcc_N	.48	.11	.12	.09	−.23†	−.34*	−.50*	.55*	
9	StroopAcc_I	.66	.21†	.18†	.19†	−.12	−.10	−.68*	.56*	.62*
10	StroopRT_DIFF_N_	.02								
11	StroopRT_DIFF_C_	.21								
12	StroopRT_RES_N_	.05								
13	StroopRT_RES_C_	.25								
14	StroopIE_DIFF_N_	.72								
15	StroopIE_DIFF_C_	.74								
16	StroopIE_RES_N_	.75								
17	StroopIE_RES_C_	.77								
18	Word-GoRT	.94								
19	Number -GoRT	.96								
20	Word-SSRT_central_	.88								
21	Word-SSRT_integration_	.89								
22	Number-SSRT_central_	.91								
23	Number-SSRT_integration_	.89								

*Note.* Stroop: MRT, mean reaction time; IE, inverse-efficiency-based scores; Acc, percentage accuracy; _C, Congruent condition; _N, Neutral condition; _I, Incongruent condition; RT, reaction-time-based scores; DIFF, difference scores; RES, regression residual scores; subscript N, Neutral baseline; subscript C, Congruent baseline. Stop-signal: GoRT, “go” mean reaction time; SSRT_central_, SSRT central; SSRT_integration_, SSRT integration. Figures rounded to two decimal places.

† *p*<0.05, two-tailed;

*significant after Bonferroni correction.

### A Latent Variable Approach

**Figure 2:**
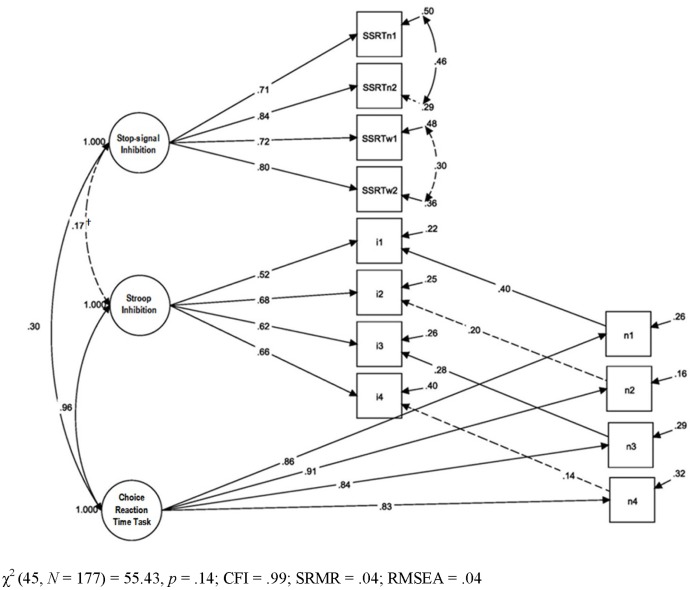
Correlation between Stroop and stop-signal inhibition: Two-factor latent variable model. SSRTn1 & 2 and SSRTw1 & 2 are Number and Word SSRT_central_ from blocks 1–2 combined and 3–4 combined, respectively; i1– i4 and n1– n4, are mean RT from blocks 1–4 of Stroop Incongruent and Neutral conditions. Standardized estimates shown. Dashed paths are insignificant at *α = *.05, two-tailed. The symbol † denotes significance at *α = *.05, one-tailed.

With the poor reliability of the derived Stroop measures, we used the component RT measures and analysed them using a latent variable approach to examine their relations with measures from the stop-signal task. We first re-examined the relationship between stop-signal and Stroop inhibition with a two-factor model corresponding to the two tasks. SSRT_central_ from the two stop-signal tasks were parcelled and modelled as four manifest indicators for stop-signal inhibition. Incongruent RT from each of the four blocks from the Stroop task served as indicators for Stroop inhibition. To take account of individual differences in simple decision time, the influence of baseline Stroop RT was modeled by regressing each incongruent indicator onto its corresponding neutral RT. These neutral items also served as indicators for a neutral or baseline choice reaction time (CRT) task latent which accounted for their inter-correlations (see Figure 2).

A more conventional way to separate the inhibitory from simple choice reaction processes in the Stroop incongruent measures might have been to cross-load the incongruent indicators onto the CRT task latent together with the neutral indicators. However, cross-loading can present challenges to the interpretation of the latents they load on [56]. We thus adopted the alternative regression approach to remove Stroop CRT task-related variance for a purer measure of the Stroop inhibitory latent construct [56].

The resulting model provided a good fit to the data, χ^2^ (45, *N* = 177) = 55.43, *p* = .14; CFI = .99; SRMR = .04; RMSEA = .04, with all indices satisfying the rule-of-thumb values [57]. All the latent variables showed significant amounts of variance. The correlation between the stop-signal and Stroop inhibition latents (*r* = .17) was only marginally significant when evaluated at *α = *.05, two-tailed, but significant when evaluated at *α = *.05, one-tailed. The high correlation (*r* = .96) between the Stroop inhibition and CRT task latents was a concern, though consistent with previous findings of high correlations between inhibitory and non-inhibitory measures from inhibitory tasks [56]. To test the possibility that the incongruent and neutral Stroop measures captured a unitary construct, we constrained the covariance between the Stroop inhibition and CRT task latents to one, and their respective covariances with the stop-signal inhibition latent to equality. The resulting model showed a significantly poorer fit, χ^2^ (46, *N* = 177) = 61.42, *p* = .06; CFI = .99; SRMR = .04; RMSEA = .04; χ^2^_diff_ (1) = 5.99, *p*<.05, favoring the prior model with separate Stroop inhibitory and CRT constructs. To test explicitly the hypothesis that the Stroop and stop-signal inhibitory measures index the same underlying construct, we constrained the covariance between the Stroop and stop-signal inhibition latents in the original model to one, and their respective covariances with the CRT task latent to equality. This model showed a significantly poorer fit than the original model, χ^2^ (46, *N* = 177) = 113.40, *p*<.01; CFI = .96; SRMR = .13; RMSEA = .09; χ^2^_diff_ (1) = 57.97, *p*<.05. Results favored an interpretation that the Stroop and stop-signal tasks measure different underlying constructs.

## Discussion

The present study explored the relationship between Stroop and stop-signal inhibition, examining whether observed correlations differ across task contexts, measure selection, and methods of derivation. Consistent with our previous finding based on Stroop and stop-signal commission errors [19], the RT data shows that poor performance on one inhibitory task does not predict poor performance on the other; the two are likely to measure different underlying constructs. However, the correlation between SSRT and Stroop commission errors suggest a close-to-moderate relationship. Participants who were slower to cancel an initiated response were also likely to make more slips when resisting responses to irrelevant-but-dominant stimulus dimensions. This makes intuitive sense: on Stroop incongruent trials, some ability to stop or inhibit fast prepotent processing before the ballistic point-of-no-return [37] is likely to be required for the slower deliberate route processes to reach completion for a correct response. It has been suggested that some common neural regions implicated across inhibitory tasks reflect a common “stop” command [58]. Findings of an interaction between Stroop congruency and stop-signal inhibitory performance in hybrid tasks have also been interpreted as reflecting overlapping mechanisms [59], [60]. Simple stopping efficiency, as indexed by the stop-signal reaction time, may be a general componential process or mechanism influencing successful inhibition in general. Alternatively, the observed relationship may reflect common proactive control mechanisms such as attentional focus or goal maintenance [30], or conflict monitoring [59]. The higher correlations observed between SSRT and Stroop errors when the tasks involved similar stimuli material (both numerical) highlight possible influences from task context.

That the relationship between stopping speed and successful Stroop inhibition does not apply to the speed of Stroop interference resolution is likely due to the more complex interplay of processes that determines the time it takes to make a correct response on Stroop incongruent trials. Accurate performance on incongruent trials is a result of relevant stimulus-response processes along the deliberate route (color-naming) and irrelevant stimulus-response and inhibitory processes along the automatic route (word-reading). Individual differences in relative automaticity between the relevant and irrelevant stimulus-response dimensions can be expected to contribute to the time taken to issue the correct response. Furthermore, the horse-race model [7] posits that inhibition in the stop-signal task takes place at the late response execution stage. On the other hand, dual-route process models of Stroop inhibition [10], [11] allow conflict resolution processes to accumulate at any stage from stimulus perception to response activation. Low correlations between Stroop and stop-signal RT measures may thus reflect the engagement of different inhibitory processes, or similar inhibitory processes at different stages: resolution of late output-stage/response-related interference in the stop-signal and a combination of early-perceptual/stimulus-level interference, intermediate-stage/cognitive interference and late output-stage/response-related interference in the Stroop [3], [61]. It has also been argued that a common inhibitory mechanism may be engaged to different extents across inhibitory tasks, in response to different levels of inhibitory demand due to differences in ancillary processes or strength of the process/response to be inhibited [27].

The low reliability of the Stroop interference RT measures may be another reason for low observed correlations. Consistent with previous studies, the present study found SSRTs to be relatively stable and reliable across variations in the primary task and estimation method. Findings provide further support for the robustness of the SSRT as a valid and reliable measure of individual differences in inhibition [62]. The Stroop measures, on the other hand, were less consistent. Variants were less highly correlated and the low reliability of the interference RT scores was problematic. Due to the high correlation of mean RTs across conditions, reliability was extremely low for the Stroop difference and regression-residual scores. True, non-artifactual correlations between measures can be obscured by low reliability in either measure as the maximum observed correlation a test can have with another variable is limited by the square root of its reliability coefficient [53]. The larger correlation observed when Stroop component scores were used in a latent variable approach, or when the comparatively more reliable IE-based scores were used instead, is consistent with the argument that observed correlations may be depressed by problems with reliability.

Differences in reliability might thus have contributed to the variation in correlations observed between different pairs of Stroop interference and SSRT measures (*r* = .00–.25). Alternatively, the variability may be due to different measures emphasizing or capturing different aspects of inhibitory performance. For instance, SSRT measured with the accuracy-based tracking algorithm may reflect influences from both speed and accuracy, while Stroop RT measures are based solely on speed. Higher correlations might hence have been observed when the Stroop interference measure also reflected influences from both speed and accuracy (i.e., IE-based scores). Nevertheless, there was still substantial variation across IE scores (*r* = .08–.25) and correlations remained marginal. However, it should also be noted that although the largest observed correlation (*r* = .25) did not reach statistical significance after correcting for multiple comparisons, its magnitude exceeds that found in some previous studies (*r* = .15–.18; [2], [16]), and would have been reported as significant if correction for multiple comparisons had not been conducted. On the other hand, many of the alternative pairs of Stroop and stop-signal RT measures would have led to findings of negligible observed correlations. The pattern of results suggests that findings on the observed relationship between Stroop and stop-signal inhibition can be influenced, to a certain extent, by variations in dependent measure derivation.

## Conclusions

Are different inhibitory constructs measured by the Stroop and stop-signal inhibition tasks? The present sample had approximately 80% power in detecting a correlation as small as *r = *.18 and 99% power in detecting a moderate-sized correlation (*r = *.30). The present findings suggest that, at least within the limits of a moderate relationship, it seems unlikely that conventional RT measures from the two tasks measure the same effect. The largest positive correlation found between the Stroop and stop-signal RT measures, in the forms they are commonly used, was a small and insignificant *r = *.08. Although the current sample size would have had insufficient power (∼28%) to detect a significant correlation this small, one needs to question its meaningfulness when such limited variance is shared between tasks purportedly measuring the same construct. It might have been that the observed correlations were depressed by a mismatch in the aspects of inhibitory control reflected or by the low reliability of the derived Stroop RT measures. However, even with a latent variable approach using raw RTs, the correlation was only slightly improved. While correlations of such magnitudes (i.e., *r = *.17) had previously been found in some studies [2], [16], measures reflecting the same competency should be expected to correlate strongly and positively. Explicit tests of a unitary-construct model favor the interpretation that the Stroop interference and stop-signal RT measures indexed different constructs. Similarly, even when the Stroop interference measures used were the more comparable (and more reliable) adjusted RT measures (i.e., IE scores), some of the observed correlations were just as small and insignificant (*r = *.08–.25). Even though the largest observed correlation surpassed that found in some previous studies, the ratio of common to unique variance between the two measures indicates way more divergence than convergence [63].

We have found no compelling evidence of a robust relationship between Stroop and stop-signal RT measures of inhibition. But what does this mean? Stroop and stop-signal RT measures may indeed be capturing different competencies. It may also still be possible that they involve the same inhibitory mechanism, but with some major differences in how the mechanism is executed (e.g., similar processes executed at different stages or to varying degrees; similar core processes confounded with different auxiliary sub-processes) or is reflected in dependent measures. However, that their behavioral measures are not strongly related poses empirical and practical problems when the tasks are often used synonymously in the literature to assess the same cognitive functions. The present data suggest that Stroop inhibitory *ability* (indexed by errors), is at least moderately related to stop-signal inhibitory *efficiency* (indexed by RT). However, inhibitory *efficiency* measured by RT scores of the two tasks should not be taken to index similar constructs or processes–a participant may be classified as a poor inhibitor when measured on one task yet do well on the other. In addition, all versions of our Stroop interference RT measures (when unadjusted by accuracy) were not significantly correlated with Stroop errors. Hence, though accuracy and RT both reflect inhibitory performance on the Stroop task, they likely reflect different competencies. The low reliability of conventional measures of Stroop interference is problematic and warrants further research in identifying a more reliable measure of Stroop inhibition.

The inverse efficiency score shows some promise with its substantially higher reliability and its attempts at reconciling speed with accuracy performance. However, its reliability is still less than satisfactory and its suitability as a measure of inhibitory performance needs to be further examined. Some doubts that have been raised about the IE score include the increased variability associated with multi-component measures; the limits to its application when there is not a high, positive correlation between RT & accuracy; and most importantly, whether dividing RT by percentage accuracy is the most appropriate way of reconciling the two aspects of performance [64]. Cognitive studies are often interested in comparing performances on conditions comprising as little as 20 to 30 trials each. A small difference in the number of errors between conditions can translate to a large difference in terms of percentage accuracy. The effect of errors on IE scores is hence not linear but accelerates with lower percentage accuracies. Consider for example, a Stroop task comprising 20 trials each of Congruent and Incongruent conditions and a participant scoring a mean Congruent RT of 600 ms, a mean Incongruent RT of 680 ms, and a 2-error difference between conditions (i.e., a.10 difference in percentage accuracy). If Congruent percentage accuracy was at 1.00, the resulting difference in IE score will be 156 ms. If Congruent percentage accuracy was at.80, the resulting difference in IE score inflates to 221 ms. RTs may thus be disproportionately adjusted, resulting in spuriously exaggerated differences between conditions [64]. Hence, examining Stroop interference in terms of IE may not be the most prudent either.

The identification of an accurate and reliable measure of Stroop inhibition warrants further research. Currently available options come with various limitations as seen in the present study that future studies will need to keep in mind when selecting dependent measures.

One way forward is suggested by the advent of the Dual Mechanisms of Control framework [33], which has generated a growing interest in examining inhibitory phenomena in terms of proactive and reactive control. More studies have begun examining other parameters in inhibitory tasks that may allow for a better delineation amongst component processes, such as non-inhibitory or “go” RT [65], [66], [67] and movement time (MT) [68], [69] as indicators of preparatory/proactive control processes. Continued efforts in this direction may lead to the development of purer measures of mechanisms/processes involved in inhibitory performance, facilitating more direct comparisons across different inhibitory paradigms..

Findings from the present study should be interpreted with a few limitations on generalizability in mind. First, the current study was conducted on adolescents to maintain comparability with its antecedent study. The focus was on examining Stroop and stop-signal measures of inhibition in this age group. However, adolescence is sometimes characterized by impulsive risk-taking behaviors which have been associated with poor inhibitory control [70]. Accompanying developmental changes in brain maturation/deterioration [71] and in response strategies such as speed-accuracy tradeoffs, performance on tasks requiring inhibition typically improves through childhood to adulthood before declining with old age [1], [50], [72], [73]. It is thus uncertain if the reported pattern of results will be observed in other age groups. Second, although efforts were made to include a nationally representative sample of Secondary 2 students, the high exclusion rate based on task engagement resulted in an underrepresentation of the lowest academic stream. Generalizability to low-achieving students may be limited.
